# Summer holidays as break-point in shaping a tannery sludge microbial community around a stable core microbiota

**DOI:** 10.1038/srep30376

**Published:** 2016-07-27

**Authors:** Cesira Giordano, Vittorio Boscaro, Giulio Munz, Gualtiero Mori, Claudia Vannini

**Affiliations:** 1 Biology Department, Protistology-Zoology Unit, University of Pisa, Via A. Volta 4, 56126, Pisa, Italy; 2 Department of Civil and Environmental Engineering, University of Florence, Via S. Marta n.3, 50139, Florence, Italy; 3 CER2CO (CEntro Ricerca Reflui Conciari) Via Arginale Ovest 8, 56020, San Romano-S. Miniato, Pisa, Italy

## Abstract

Recently, several investigations focused on the discovery of a bacterial consortium shared among different wastewater treatment plants (WWTPs). Nevertheless, the definition of a core microbiota over time represents the necessary counterpart in order to unravel the dynamics of bacterial communities in these environments. Here we performed a monthly survey on the bacterial community of a consortial industrial plant. Objectives of this study were: (1) to identify a core microbiota constant over time; (2) to evaluate the temporal dynamics of the community during one year. A conspicuous and diversified core microbiota is constituted by operational taxonomic units which are present throughout the year in the plant. Community composition data confirm that the presence and abundance of bacteria in WWTPs is highly consistent at high taxonomic level. Our results indicate however a difference in microbial community structure between two groups of samples, identifying the summer holiday period as the break-point. Changes in the structure of the microbial community occur otherwise gradually, one month after another. Further studies will clarify how the size and diversity of the core microbiota could affect the observed dynamics.

Waste Water Treatment Plants (WWTPs) contain highly diverse and dynamic microbial consortia, where bacteria are the dominant organisms responsible for the removal of pollutants. Many studies highlighted the importance of the bacterial community dynamics for the stability of wastewater treatments^1^,^2^, although variation in bacterial composition may not always cause changes in wastewater treatment performances^3^,^4^. The study of microbial communities is actually important not only for an improvement of the treatment efficiency, but also as a fundamental prerequisite for future exploitations of energy and biomaterial resources from WWTPs^5^. Moreover, these communities represent an ideal model system for defining properties of microbial consortia in a monitored and highly-controlled environment^6^,^7^.

Recently, many investigations focused on the discovery of a bacterial consortium shared among different WWTPs. Several studies showed that bacterial communities of municipal WWTP reactors operating under diverse configurations at different geographic locations are highly similar at least at high taxonomic ranks such as phyla^8^,^9^,^10^,^11^. A core community of abundant genus-like Operational Taxonomic Units (OTUs) was also described for 13 different Danish WWTPs^12^,^13^ and similar results were found for 9 different Chinese WWTPs^14^. Attempts in this sense have been also recently performed on an industrial WWTP microbial community^15^. If the definition of a core community largely conserved across plants of different geographical regions and with different operational mode is certainly relevant for refining further investigations, time-series studies on WWTP microbial communities are the essential counterpart in order to depict a complete scenario. Indeed, only an integration between the two complementary aspects, the core microbiota over time and the core microbiota over space, will potentially allow a clear definition of the WWTP community nature. Time-series studies are necessary for a complete understanding of microbial dynamics^6^,^16^. Such an assumption has been supported also for WWTP communities^17^,^18^.

In the last years new technologies have been developed, such as the so-called next-generation high-throughput sequencing methods, which can elucidate the characteristics of microbial communities more completely and accurately^19^,^20^,^21^. Among these techniques, 16S rRNA gene amplicon sequencing by Illumina MiSeq is among the most recent ones and, although effective in the study of microbial communities from several different systems^22^,^23^,^24^,^25^, it has not yet been extensively used on WWTPs^26^,^27^,^28^.

In the present study, we performed a time-series study on the bacterial community of an industrial WWTP using Illumina MiSeq technology. The main objectives of this study were: (a) to identify a core microbiota of the plant constant over time and potentially representing its structuring body; (b) to evaluate the temporal dynamics of the entire bacterial community during one year by a monthly monitoring.

## Results

### Plant functioning and samples

The biological section of the wastewater treatment plant (WWTP) is constituted by a predenitrification and a nitrification-oxidation tank with an internal recirculation of 8 times the influent flow rate. The Dissolved Oxygen (DO) is controlled in the aerobic tanks at 1.8 ± 0.4 mg/l during the year. Table 1 summarizes the values of abiotic data measured over the monitored period for the nitrification tank effluent from which 12 samples were collected monthly. The plant treats both civil and industrial wastewater produced in the local tannery district. Incoming wastewater flow rates are also reported. For what concerns data of the wastewater influent, the average values over the entire period for COD, total nitrogen, chlorides and sulphates were, respectively: 5273.6 ± 1227.5 mg/l, 542.3 ± 130.5 mg/l, 6092.0 ± 1453.2 mg/l, 2378.0 ± 595.9 mg/l.

### Bacterial community composition

**Figure 1:**
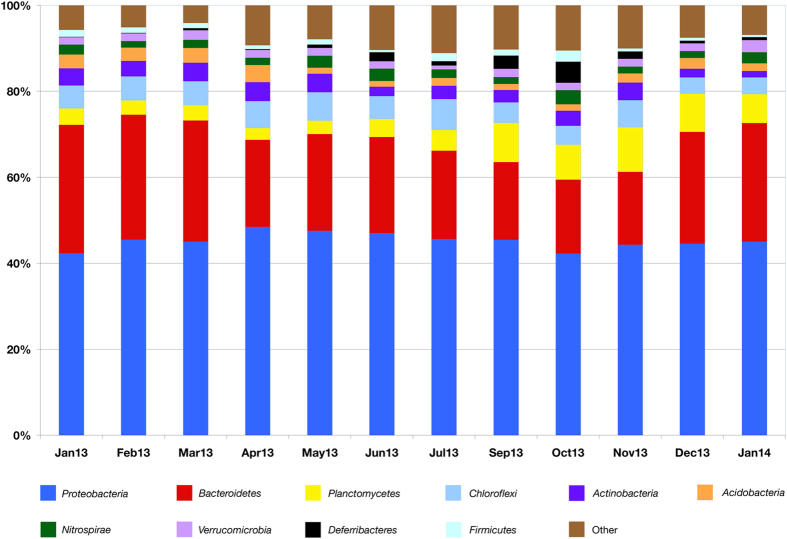
Bacterial community composition at the phylum level. Phyla with an average abundance lower than 1% are grouped in “Other”.

A total of 12,429,502 reads with a length of 300 bp were overlapped to assemble 4,855,990 contigs. After denoising, chimera filtering, and eukaryotic sequences removal, the prokaryotic library was composed by 3,874,044 contigs (average number per sample 322,837 ± 139,940), clustered in 5,029 Operational Taxonomic Units (OTUs), grouped with a 97% similarity threshold. A total of 40 phyla were found, and 10 of them were identified as major phyla (average abundance >1%). As shown in Fig. 1, *Proteobacteria* was the most abundant phylum in all samples, accounting for 45.2% ± 1.8% (average percentage of contigs per sample). The other dominant phyla were *Bacteroidetes* (23.2% ± 4.5%), *Planctomycetes* (5.7% ± 2.6%), *Chloroflexi* (5.4% ± 1.0%), *Actinobacteria* (3.3% ± 0.9%), *Acidobacteria* (2.3% ± 0.9%), *Nitrospirae* (2.1% ± 0.6%), *Verrucomicrobia* (1.8% ± 0.4%), *Deferribacteres* (1.3% ± 1.4%), and *Firmicutes* (1.2% ± 0.6%). The retrieved average number of observed OTUs per sample is 2,933 ± 73, with a minimum of 2,793 (sample of September 2013) and a maximum of 3,032 (sample of April 2013). The average value of the Shannon diversity index is 7.97 ± 0.02, with a minimum of 7.92 (sample of September 2013) and a maximum of 8.01 (sample of April 2013).

**Figure 2:**
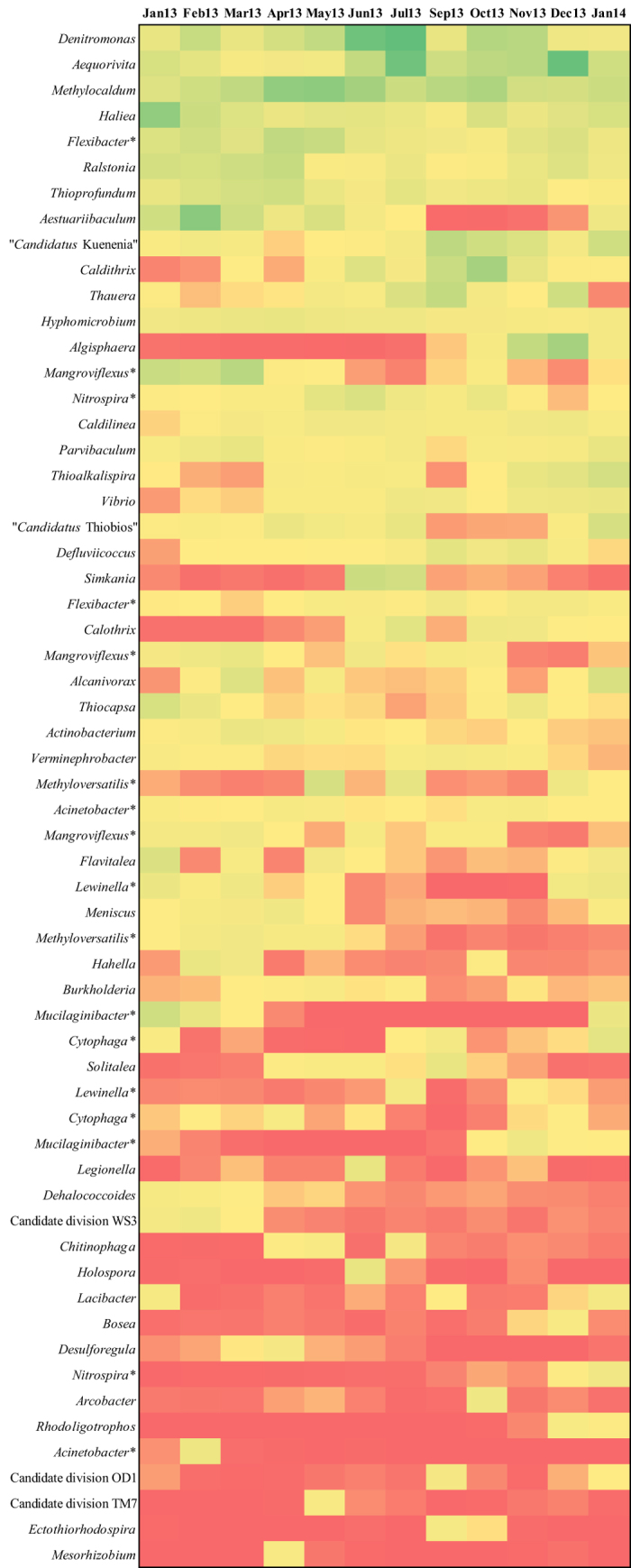
Heat map of the top 20 most abundant OTUs in each sample, classified at the genus level. Green and red indicate high and low relative abundance, respectively. (*) Genus represented by more than one OTU.

The top 20 most abundant OTUs in each sample were then selected obtaining a list of 60 OTUs, classified at the genus level (Fig. 2). Two genera of Gram negative bacteria, *Denitromonas*, comprising denitrifying bacteria (3.94% ± 2.30%), and *Aequorivita* (3.84% ± 2.23%), included in the family *Flavobacteriaceae*, were found as the two most abundant genera, followed by *Haliea* (2.03% ± 1.25%), included in the family *Alteromonadaceae*, and *Flexibacter* (1.96% ± 0.79%), comprising bulking bacteria^29^ of the family *Flexibacteriaceae*.

### Temporal variation in bacterial community structure

**Figure 3:**
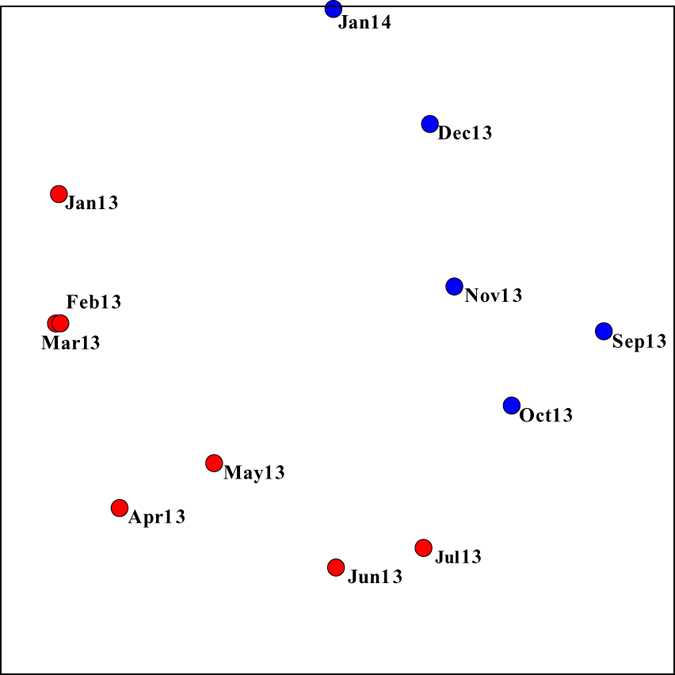
Non-metric Multi-Dimensional Scaling (nmMDS), performed on relative abundance data of the total observed OTUs. Stress value: 0.08.

The temporal dynamics of the entire bacterial community was evaluated using nmMDS ordination (Fig. 3) and the ANOSIM test, performed on relative abundance data of the total observed OTUs. The arrangement of samples in the graph does not indicate unambiguously a pronounced and obvious separation of sample groups. However, samples could be grouped according to two different patterns: January 2013-March 2013, April 2013-July 2013, September 2013-January 2014 (three groups) or January 2013-July 2013 and September 2013-January 2014 (two groups). While the first hypothesis was not supported by results of the ANOSIM test (p > 0.05), the separation in two distinct groups was supported by a significative result (p < 0.05). Differences between samples taken in colder months (January, February, March, November, December) and those taken in hotter months (April, May, June, July, September, October) were not statistically significant by ANOSIM analysis (p > 0.05). Nevertheless, an increase in similarity distances is observed in samples from January to July 2013 and then a reverse trend is shown by samples from September 2013 to January 2014 (Fig. 3). The Shannon diversity index indicates a slight increase in diversity after the transition from the first period (7.74 ± 0.06, January to July 2013) to the second period (8.30 ± 0.08, September 2013 to January 2014). This difference is statistically significant as confirmed by ANOSIM (p < 0.05).

A heat map of the 20 most abundant OTUs, classified at the level of genus, in each sample was also calculated (Fig. 2). Among these, bacteria implicated in denitrification, such as *Caldithrix* spp.^30^, or in the formation of biofilm among activated sludge flocks, such as *Thauera* spp.^31^, increased their relative abundance after the summer. On the other side, other genera involved in denitrification, such as *Denitromonas*^32^ or *Hyphomicrobium*^29^, and nitrifiers, such as *Nitrospira* spp.^33^, decreased their relative abundance in September. Considering the whole number of retrieved OTUs, 24 new taxa appeared in September and remained in the plant in the following months, such as filamentous bacteria belonging to the genera *Caldilinea*^34^ and *Acinetobacter*^35^,^36^; nitrogen-fixing bacteria of the genus *Azoarcus*^37^; denitrifying bacteria belonging to the family *Hyphomicrobiaceae*^38^. On the other hand, 29 OTUs, which were present from January to July, disappeared completely from September; of note are ammonia-oxidizing bacteria of the family *Nitrosomonadaceae*^39^; denitrifying bacteria belonging to the genus *Flexibacter*^40^; and members of the family *Sandaracinaceae*, involved in starch degradation^41^.

### Influence of environmental variables on bacterial community structure

**Figure 4:**
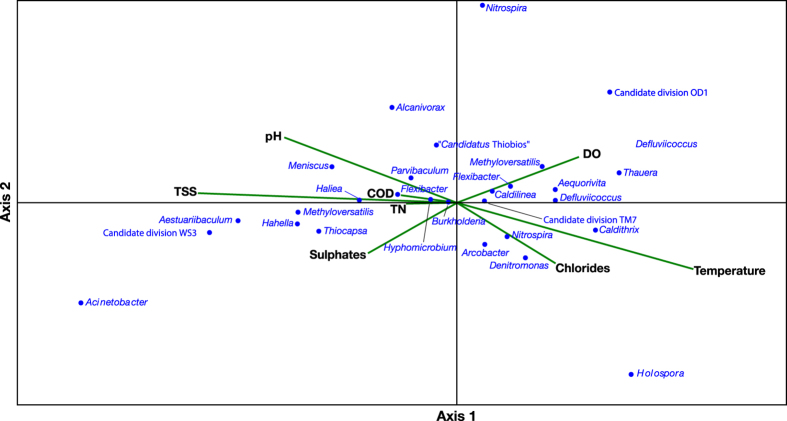
Canonical Correspondence Analysis (CCA) performed on the most represented OTUs. Percent variation explained: 39.9% (axis 1), 18.2% (axis 2). COD: Chemical Oxigen Demand; DO: Dissolved Oxygen; TN: Total Nitrogen; TSS: Total Suspended Solids.

Canonical Correspondence Analysis (CCA) was performed to determine the major environmental variables influencing the relative abundance of the 60 most represented OTUs (Fig. 4). Eight parameters were used in the CCA biplot: temperature, chemical oxygen demand (COD), total nitrogen (TN), total suspended solids (TSS), dissolved oxygen, pH, chlorides and sulphates. The length of an environmental parameter arrow in the ordination plot indicates the strength of the relationship of that parameter to community composition. Temperature and TSS, which, in turn, can be determined by different factors like the solid retention time or incoming load, are represented by the longest arrows. A BIO-ENV analysis was then performed in order to identify the combination of abiotic parameters that best explained changes in the microbial community. Again, the combined effect of temperature and TSS was shown to be the most suitable to explain the observed changes with a Rho value of 0.569 and a P value of 0.03.

### Core microbiota and unique OTUs

Comparative analysis revealed a core microbiota across the 12 samples. Among the 5029 OTUs, 1218 (accounting for 24.2%) were shared by all 12 samples, constituting a core maintained over the whole year. A total of 2141 OTUs (accounting for 42.6%) were shared by at least 10 samples. Mean relative abundance of the core OTUs is 87.4% ± 2.8%, with a maximum in the sample of July 2013 (93.2%) and a minimum in the sample of January 2014 (81.0%). 52 out of the 60 more abundant OTUs (i.e. the group constituted by the 20 more abundant OTUs in each sample) are included in the core.

There were 49 unique OTUs detected in only one sample, accounting for 0.97% of the total. Among these, a conspicuous fraction (32.7%) appeared only in the September sample.

## Discussion

As recently reported^6^,^16^, it is possible to highlight different properties of a system depending on the performed frequency of sampling and to the used methods. Our one-year monthly survey by 16S rRNA gene amplicon Illumina sequencing allowed us to describe composition and dynamics of the bacterial community and to unravel at least some of its key drivers.

Regarding the community composition, *Proteobacteria* was the most represented phylum, followed by *Bacteroidetes*. With a few exceptions (for example Munck and colleagues^42^), similar results were reported in previous studies, which found that these two phyla actually are the dominant bacterial groups in WWTPs^9^,^10^,^11^,^15^,^43^,^44^. The other recovered major phyla (percentage >1%) were also widespread in other WWTP facilities^14^,^15^,^29^,^30^,^31^,^45^. Our data on the presence and relative abundance of bacterial phyla are thus in agreement with previous findings, confirming that the distribution of bacteria in most WWTPs is highly consistent at high taxonomic level.

Comparative analysis revealed the presence of a conspicuous and diversified core microbiota constituted by OTUs which are present throughout the year in the plant. We actually identified for the first time the constant core microbiota in a plant treating industrial wastewater and we also showed that such microbiota has a mean relative abundance of 87.4%, which represents a very large fraction of the total biomass of the plant, surely playing a major role in determining biomass behavior. Similar data were actually found by Ju and Zhang^46^, who showed that a persistent fraction of the biomass (present in ≥80% of collected months) accounted for 76.6% in abundance in a municipal WWTP. Therefore, the presence of a stable and substantial core microbiota over time could represent a feature shared even by plants operating in different conditions. This information is extremely useful to define the steadily present community members for e.g. to build predictive models. Other studies focused more on a bacterial core shared by different WWTPs^8^,^9^,^10^. In both instances, only half of the information is revealed. The definition of the spatial and temporal core microbiota is an essential key to deeply understand the dynamics of the microbial community and the possible trends, periodicity or irregularities that are generated. Moreover, it has been previously hypothesized that, for each plant, a core community could display higher stability and resilience, playing a major role in determining community functions over time^1^. Further studies performed on different plants and in different conditions will show differences and similarities in the composition and abundance of the core microbiota over time, revealing its degree of importance in determining biomass dynamics in WWTPs.

Our one-year recording of data indicates a difference in microbial community structure between samples collected from January 2013 to July 2013 and those collected from September 2013 to January 2014. Even if not sharp in the nmMDS ordination, the separation between the two groups was confirmed by the significance of the ANOSIM test. Data of presence/absence of OTUs and the heat map of most abundant OTUs indicate August as the break-point, too. In September, a decrease in abundance of genera such as *Denitromonas* and *Nitrospira* was observed, followed by the growth of different bacteria, such as *Defluviicoccus* spp., *Caldithrix* spp. and *Thauera* spp. The transient appearance in September of the highest fraction of unique OTUs also indicates the occurrence of a perturbation that is then somehow softened. August is traditionally the period for summer vacation in Italy, and during this month most tanneries, whose wastewater is collected to the plant, close. As a consequence, the industrial flow rate is drastically reduced (see Table 1). Therefore, the influent is characterized by a different composition (i.e. reduced content in organic compounds, sulfates and chlorides), resulting in a conspicuous change of working conditions in this period of the year. Indeed, combined data from CCA and heat map highlight how, for example, genera mainly present at high values of COD, e.g. *Flexibacter, Parvibaculum, Methyloversatilis* and *Haliea* were negatively affected by the changes occurred in August. Further studies could clarify if, in the whole community, different trends are displayed by different functional groups.

A number of studies, performed with a seasonal sampling, pointed to a significant difference in bacterial communities between winter and summer samples in WWTPs (see for example refs 3 and 18). Our samples were collected monthly, while many previous studies performed in order to test the influence of seasonality use a lower sampling frequency (see for example refs 3 e and 47). Stated that a one-year time-series study could be not sufficient for assessing the influence of this aspect, the lack of significative difference between samples of the colder and hotter months could be also due to the adopted sampling scheme. Similar results were obtained by Ju and Zhang^46^, who also found no effect of seasonality after performing a time-series study by monthly collection of samples in a municipal WWTP. Indeed, our study pointed out how changes in the microbial community structure seem to occur gradually one month after another. This pattern could be explained by an influence of abiotic parameters which gradually change during the whole year. According to this view, the importance of temperature (together with SST) was assessed by the results of both CCA and BIO-ENV analysis. Indeed, temperature is already known to heavily affect the bacterial growth and to influence the dynamics of the bacterial community in WWTPs^14^,^17^,^48^.

Our time-series data indicate that, after a period of acclimatization in September, the composition of the bacterial community start changing with a different path. A monthly monitoring over more years would be necessary in the future to assess if this difference indicates a regular trend of shifting to an alternative state as observed in previous studies^46^ and how the size and diversity of the core microbiota could affect this dynamics.

## Methods

### Sample collection

Twelve samples of activated sludge were collected monthly, from January 2013 to January 2014, from the nitrification tank effluent of a full scale wastewater treatment plant (WWTP) managed and operated by the Cuoiodepur S.p.A. (San Romano, Pisa, Italy). The plant treats both civil and industrial wastewater produced in the local tannery district. The configuration of the process in the biological section comprises predenitrification, nitrification and separation. Abiotic parameters of the sampled effluent such as temperature, pH, chemical oxygen demand (COD), total nitrogen (TN), total suspended solids (TSS), chlorides and sulphates were constantly monitored throughout the studied period. Abiotic parameters of the influent were monitored, too, and COD, total nitrogen, chorides and sulphates were used for CCA analysis.

### DNA extraction, PCR amplification and sequencing

Two ml of each sample were stored in 70% (v/v) ethanol at −20 °C for DNA extraction. Total genomic DNA was extracted using PowerSoil^®^ DNA Isolation Kit (MoBio), according to the manufacturer’s instructions. The concentration and purity of the extracted DNA was determined by Qubit^®^ 2.0 Fluorometer and the dsDNA HS Assay Kit (Life Technologies); each DNA solution was then diluted to a concentration of 5 ng/μl.

PCR amplification (using the BioRad C1000 Thermal Cycler and the KAPA HiFi HotStart Ready Mix) was carried out on normalized DNA solutions using the primer set for the V3–V4 regions of the 16S rRNA gene reported by Klindworth and colleagues^49^ as the most promising bacterial primer pair (PCR forward primer 5′-CCTACGGGNGGCWGCAG-3′ and PCR reverse primers 5′-GACTACHVGGGTATCTAATCC-3′). The Illumina forward and reverse overhang adapter sequences added to each primer were 5′-TCGTCGGCAGCGTCAGATGTGTATAAGAGACAG-3′ and 5′-GTCTCGTGGGCTCGGAGATGTGTATAAGAGACAG-3′, respectively (Illlumina prot., Part # 15044223, Rev. B). Two separated PCR reactions were performed, the Amplicon PCR and the Index PCR, following the Illumina protocol. The amplicons were purified after each step using the AMPure XP beads (Beckman Coulter). The resulting libraries were normalized and pooled. The pooled sample was sequenced by BMR Genomics (Padova, Italy) on a MiSeq platform (2 × 300 paired-end sequencing with MiSeq Reagent Kit v3).

### High-throughput DNA sequencing analyses

Raw reads were analyzed using the Quantitative Insights Into Microbial Ecology 1.8 (QIIME) software package^50^. FastQC^51^ was used to assess data quality. Merged sequences (contigs) shorter than 250 bases after primer trimming with the Cutadapt software^52^ and those that did not contain recognizable primer sequences (allowing up to 10% mismatches) were discarded. OTUs were grouped with a 97% similarity threshold using the SILVA 111 database as reference^53^; sequences that did not match any reference entry were grouped in *de novo* OTUs. The most common sequence in each OTU was selected as representative. Taxonomic classification down to the phylum, class, order, family and genus level was performed using QIIME via the SILVA 111 database. Putative chimeras identified by Uchime^54^ were removed from the analyses, as well as sequences of eukaryotes (removed by QIIME). Finally, for each sample, OTUs represented by less than 10 sequences were discarded as “noise”. Data processing following the OTUs search step was performed both on QIIME and the PAleontological STatistic (PAST) software^55^.

### Statistical analyses

Relative abundances were calculated (measured as the number of contigs) and a heat map analysis was performed. A heat map of the 20 most abundant OTUs, classified at the level of genus, in each sample was also calculated. QIIME was used to estimate beta diversity. The Shannon index and the number of observed OTUs were calculated to assess the community richness. Similarities among samples were assessed graphically by the ordination method of non-metric Multi-Dimensional Scaling (nmMDS), based on Bray–Curtis distances. The ANalysis Of SIMilarity (ANOSIM) was used to evaluate the statistical significance of observed differences between groups of data. To investigate the effect of both environmental and operative parameters on the most represented OTUs, Canonical Correspondence Analysis (CCA) was performed using the software PAST^62^. A BEST/BIO-ENV analysis was carried out using the software PRIMER-E v6 (PRIMER-E Ltd, Ivybridge, UK) with Spearman and 999 permutations; Euclidean distances were used for normalized abiotic parameters and Bray-Curtis distances for abundance data. To visualize unique OTUs as well as OTUs shared among different samples the Cytoscape software was used^56^. All samples were normalized randomly extracting an equal number of sequences from each.

## Additional Information

**How to cite this article**: Giordano, C. *et al.* Summer holidays as break-point in shaping a tannery sludge microbial community around a stable core microbiota. *Sci. Rep.* **6**, 30376; doi: 10.1038/srep30376 (2016).

## Figures and Tables

**Table 1:** Abiotic parameters of the nitrification tank effluent.

	T* (°C)	pH*	TSS* (mg/l)	COD (mg/l)	N tot (mg/l)	Chlorides (mg/l)	Sulphates (mg/l)	SVI (ml/g)	Domestic wastewater flow rate (m^3^/d)	Industrial wastewater flow rate (m^3^/d)
January 2013	20.0 ± 1.6	7.2 ± 0.1	9552.4 ± 409.7	335.8 ± 65.1	25.5 ± 5.2	2713.4 ± 599.9	1555.2 ± 416.0	152.9 ± 31.2	3401 ± 917	6610 ± 1779
February 2013	21.8 ± 0.8	7.3 ± 0.1	9487.4 ± 580.5	385.2 ± 27.7	28.1 ± 3.9	3297.6 ± 163.0	1892.0 ± 109.3	141.5 ± 23.1	3515 ± 752	7033 ± 1011
March 2013	22.9 ± 1.1	7.2 ± 0.1	10739.0 ± 728.8	347.8 ± 33.9	23.9 ± 3.5	2816.0 ± 299.0	1618.6 ± 160.0	108.2 ± 21.2	3405 ± 1105	8299 ± 1721
April 2013	25.0 ± 1.7	7.2 ± 0.1	9237.9 ± 438.4	373.9 ± 45.1	24.7 ± 3.1	2800.9 ± 322.4	1547.2 ± 163.2	111.1 ± 15.3	3741 ± 856	7358 ± 1444
May 2013	28.2 ± 1.2	7.1 ± 0.2	8691.8 ± 411.1	416.7 ± 38.3	23.6 ± 2.0	2828.0 ± 247.0	1583.7 ± 163.0	115.0 ± 15.5	4473 ± 1124	6740 ± 1426
June 2013	30.4 ± 1.6	7.0 ± 0.1	7715.0 ± 354.7	428.0 ± 23.1	22.8 ± 2.3	3014.9 ± 166.9	1541.6 ± 77.9	126.5 ± 19.1	3398 ± 875	6141 ± 1099
July 2013	34.0 ± 1.5	7.1 ± 0.2	7094.8 ± 700.0	514.8 ± 60.8	27.0 ± 4.4	3321.6 ± 175.0	1709.7 ± 101.4	119.1 ± 19.6	3451 ± 935	5811 ± 1431
August 2013	31.0 ± 3.2	7.2 ± 0.2	5840.8 ± 1487.2	333.1 ± 178.5	24.7 ± 6.7	1890.1 ± 1066.9	1124.7 ± 727.2	146.2 ± 43.1	4700 ± 1512	1520 ± 998
September 2013	30.6 ± 0.8	7.1 ± 0.1	6774.5 ± 1186.9	357.5 ± 63.3	28.5 ± 6.6	2760.3 ± 658.5	1643.3 ± 68.5	124.0 ± 25.1	4052 ± 1217	6016 ± 1908
October 2013	30.2 ± 0.5	7.0 ± 0.2	9213.6 ± 689.1	418.5 ± 22.1	28.6 ± 5.5	3124.8 ± 184.0	1576.1 ± 96.7	114.7 ± 14.7	3148 ± 985	7136 ± 1761
November 2013	27.5 ± 1.3	7.0 ± 0.1	8105.0 ± 262.8	439.6 ± 28.4	30.9 ± 4.1	3049.8 ± 245.8	1560.2 ± 163.2	109.1 ± 15.9	3581 ± 762	6420 ± 1387
December 2013	24.0 ± 1.8	7.1 ± 0.1	8356.5 ± 643.7	456.9 ± 27.1	30.4 ± 5.2	3146.8 ± 326.6	1591.3 ± 126.6	114.2 ± 19.3	3679 ± 832	5551 ± 1601
January 2014	20.4 ± 2.6	7.1 ± 0.1	9723.0 ± 585.2	324.0 ± 56.4	22.2 ± 4.6	2515.5 ± 629.8	1394.4 ± 417.9	112.4 ± 9.5	3396 ± 1647	7285 ± 2692

Wastewater flow rates are also reported.

^*^measured inside the tank.
